# IL6 and BCL3 Expression Are Potential Biomarkers in Esophageal Squamous Cell Carcinoma

**DOI:** 10.3389/fonc.2021.722417

**Published:** 2021-08-04

**Authors:** Sheila Coelho Soares-Lima, Isabela Martins Gonzaga, Diego Camuzi, Pedro Nicolau-Neto, Raissa Vieira da Silva, Simone Guaraldi, Maria Aparecida Ferreira, Hector Hernandez-Vargas, Zdenko Herceg, Luis Felipe Ribeiro Pinto

**Affiliations:** ^1^Programa de Carcinogênese Molecular, Instituto Nacional de Câncer (INCA), Rio de Janeiro, Brazil; ^2^Seção de Endoscopia, Instituto Nacional de Câncer (INCA), Rio de Janeiro, Brazil; ^3^Cancer Research Centre of Lyon (CRCL), Inserm U 1052, CNRS UMR 5286, Centre Léon Bérard, Université de Lyon, Lyon, France; ^4^Epigenetics Group, Mechanisms of Carcinogenesis Section, International Agency for Research on Cancer (IARC), Lyon, France

**Keywords:** esophageal squamous cell carcinoma (ESCC), BCL3, IL6, biomarker, therapeutic target, diagnosis

## Abstract

Esophageal squamous cell carcinoma (ESCC) ranks among the most lethal tumors worldwide, as a consequence of late detection and poor treatment response, evidencing the need for diagnosis anticipation and new therapeutic targets. First, we investigated the IL6 gene and protein expression in the esophagus of individuals without esophageal disorders (healthy), ESCC, and non-tumoral surrounding tissue (NTST). Our results showed that IL6 mRNA and protein expression is upregulated in tumor cells relative to NTST. In the TCGA dataset, we identified a set of genes whose expression was correlated with *IL6* mRNA levels, including the antiapoptotic gene *BCL3*. By using an immortalized esophageal cell line, we confirmed that IL6 was capable of inducing *BCL3* expression in esophageal cells. BCL3 mRNA and protein are overexpressed in ESCC and NTST compared to healthy esophagus, and *BCL3* mRNA could distinguish the morphologically normal samples (healthy and NTST) with 100% sensitivity and 95.12% specificity. The spatial intratumoral heterogeneity of both *IL6* and *BCL3* expression was evaluated, corroborating *IL6* upregulation throughout the tumor, while tumor and NTST showed a consistent increase of *BCL3* expression relative to the healthy esophagus. Our study shows that IL6 overexpression seems to be a key event in ESCC carcinogenesis, contributing to ESCC through a homogeneous antiapoptotic signalling *via* BCL3 overexpression, thus suggesting anti-IL6 therapies to be further considered for ESCC treatment. Finally, our data support the use of *BCL3* mRNA expression as a potential biomarker for ESCC detection.

## Introduction

Esophageal squamous cell carcinoma (ESCC) accounts for the majority of esophageal tumors around the world, with a significant impact on public health since this cancer is the seventh most common type of cancer worldwide, affecting mainly low and middle-income countries. Furthermore, ESCC presents a poor prognosis, with an overall survival between 10 and 20%, mainly due to late-stage diagnosis (1, 2). Therefore, the development of biomarkers that can help ESCC early diagnosis is extremely important. Chronic irritation and inflammation of the esophageal epithelium have been correlated with an increased risk of ESCC development. Complementary, genome-wide DNA methylation profiling of esophageal squamous cell carcinoma carried out by our group showed that genes involved in the inflammatory response are commonly altered by DNA methylation (3, 4). Among them, the hypomethylation of interleukin-6 (*IL6*) promoter suggests an upregulation of this cytokine in ESCC, corroborating previous studies (5–7).

IL6 is a pleiotropic cytokine first described for its role in the induction of B cell maturation into antibody-producing cells (8). IL6 binds to the IL6 receptor that can be found anchored in the cell membrane (mbIL-6R) or in its soluble form (sIL-6R). The classical signaling is triggered by IL6 binding to mbIL-6R and the consequent dimerization of gp130. This leads to the activation of cytoplasmic tyrosine kinases that, in turn, activate the JAK/STAT axis. Alternatively, in the so-called trans-signaling, IL6 binds to sIL-6R and activates pro-inflammatory pathways, even in cells that do not express mbIL-6R. Among IL6 transcriptional targets, many genes involved in the different cancer hallmarks have been described (9). In ESCC, for example, a role for IL6 in inhibiting apoptosis through *MCL1* induction has been described (5). But, other transcriptional targets and the putative systemic effects of IL6 have not been further explored in this tumor type.

Therefore, this study aimed to evaluate *IL6* dysregulation in ESCC and to identify potential targets that could contribute to tumor phenotypes. With this, we showed *BCL3* overexpression, already detected in the non-transformed esophageal epithelium of ESCC patients, that presents the potential to be used as a detection biomarker of the cancerization field in the esophagus. Furthermore, the IL6 signaling pathway might represent an alternative therapeutic target for these patients.

## Material and Methods

### Human Samples

A total of 77 patients with a histologically confirmed diagnosis of ESCC at Instituto Nacional de Câncer (INCA, Rio de Janeiro, Brazil), Hospital Universitário Pedro Ernesto (HUPE, Rio de Janeiro, Brazil) and Hospital das Clínicas de Porto Alegre (HCPA, Porto Alegre, Brazil) were included in this study. Frozen tissue samples were obtained from tumor and tumor-surrounding mucosa (histologically normal tissue, collected 5 cm from tumor border), and, at the moment of the biopsy, patients had not undergone any chemotherapy or radiotherapy treatments. Frozen tissue samples were also obtained from individuals without esophageal disorders (n = 68) submitted to routine endoscopic examination, not related to cancer or esophageal disorders, at HUPE. Each individual donated three biopsies, one from each third of the esophagus (superior, middle, and inferior). All individuals signed informed consent, and information was obtained from medical records and/or using a standardized questionnaire, including data on tobacco smoking and alcohol drinking. Individuals were classified as ever smokers when they smoked at least one cigarette per day for over one year. Similarly, individuals were classified as ever drinkers when they drank alcoholic beverages at least twice a week for over one year (patient’s characteristics are shown in **Table 1**).

**Table 1:** Characteristics of the individuals included in the study.

	Individuals without esophageal disorders*	ESCC patients*
Total number	68	77
Age		
Median (min-max)	57 (18-85)	59 (39-77)
Gender		
Female	43 (69%)	20 (26%)
Male	19 (31%)	57 (74%)
Smoking Status		
Never	38 (62%)	1 (2%)
Ever	23 (38%)	45 (98%)
Drinking Status		
Never	29 (47%)	6 (14%)
Ever	33 (53%)	36 (86%)
Esophageal tumor central location		
Proximal	NA	8 (10%)
Middle	NA	59 (71%)
Distal	NA	16 (19%)
Tumor Differentiation		
*in situ*	NA	1 (1%)
Well	NA	0 (0%)
Moderately	NA	55 (76%)
Poorly	NA	16 (22%)
Tumor Stage		
I-II	NA	12 (29%)
III-IV	NA	34 (71%)
Type of analysis performed		
mRNA expression	41 (45%)	39 (51%)
Protein expression	22 (24%)	38 (49%)

*Numbers may vary due to missing data. NA, not applicable.

For the analysis of intratumor heterogeneity, biopsies were collected from five patients submitted to endoscopy at INCA. Two fragments (profound and superficial) were collected from three different regions of the tumor mass: proximal, medial, and distal areas. Also, two biopsies of adjacent non-tumor tissue were collected 5 cm from the tumor border whenever possible, from the proximal and distal esophagus (**Figure 3A**). The study proposal was approved by the Ethics Committees of the institutions involved and was carried out according to the Helsinki Declaration.

### mRNA Expression Analysis

RNeasy Micro Kit (Qiagen) was used to extract total RNA from matched tumor and surrounding tissues from 39 ESCC patients and esophageal mucosa from 46 individuals without cancer. Total RNA from cell lines was extracted using the TRIzol^®^ reagent (Invitrogen). Five hundred nanograms of total RNA were used in reverse transcription (RT) reactions using SuperScript II Reverse Transcriptase, according to the manufacturer’s instructions (Life Technologies). The following primers were used in quantitative PCR (qPCR): *BCL3* forward - 5’ CGGAGCCTTACTGCCTTTGT 3’, *BCL3* reverse - 5’ GCCATGGCGATGTCAGCAGA 3’, *IL6* forward - 5’ GACCGAAGGCGCTTGTGGA 3’, *IL6* reverse - 5’ CTCATTCTGCCCTCGAGCC 3’, *GAPDH* forward - 5’ CAACAGCCTCAAGATCATCAGCAA 3’, *GAPDH* reverse - 5’ AGTGATGGCATGGACTGTGGTCAT 3’, *HPRT1* forward - 5’ CATTGTAGCCCTCTGTGTGC 3’ and *HPRT1* reverse - 5’ CACTATTTCTATTCAGTGCTTTGATGT 3’. All reactions were performed in a Rotor-Gene Q system (Qiagen) and consisted of 5.0 μL of QuantiFast SYBR Green PCR Mix 2X (Qiagen), 10 pmol of each primer and 1 μL of cDNA diluted 10 times in sterile deionized water, in a final volume of 10 μL. The thermal cycling program consisted of an initial denaturation for 5 min at 95°C, followed by 40 cycles of 5 s at 95°C and 10 s at 60°C. At the end of the cycling, dissociation curves were added to inspect the formation of nonspecific products and contamination. All analyses were done in triplicates, and the mean was used for further calculations. *BCL3* and *IL6* mRNA expression were calculated by the ΔCt method, using *GAPDH* or *HPRT1* as the housekeeping gene. The data was presented as 2^−ΔCt^. Undetermined Ct values for the amplification of target genes were set to 40 to allow further comparisons.

### Immunohistochemistry

Immunohistochemistry was performed on paraffin sections of 38 ESCC cases, including eight cases with non-tumor surrounding tissue, and 22 healthy controls. For antigen retrieval, sections were incubated in a pressure cooker while submerged in citrate buffer, pH 6.0 for BCL3 staining and EDTA buffer, pH 8.0 for IL6 detection. Sections with 3 µm were then incubated in 3% hydrogen peroxide for 20 min and Protein Block solution for 30 min (Dako^®^, Denmark) before the incubation with the primary antibody against BCL3 (Abcam^®^ – ab49470) or IL6(Abcam^®^ – ab6672), overnight at 4°C. Detection and staining were performed with the Novolink™ Polymer Detection System (Leica Biosystems, UK). Sections were counterstained with Harris’ hematoxylin. FFPE healthy tonsil was used as a positive control of BCL3 expression and lymph node as a positive control to IL6 detection. In the negative control, the primary antibody was replaced with the antibody diluent solution.

### Cell Line and IL6 Treatment

HET-1A, a normal esophagus epithelial cell line immortalized with SV40 large T antigen, was purchased from ATCC and grown in bronchial epithelial cell growth medium (BEGM) containing all supplements provided by the manufacturer (Lonza) in flasks pre-coated with a mixture of 0.01 mg/mL fibronectin, 0.03 mg/mL bovine collagen type I and 0.01 mg/mL bovine serum albumin dissolved in the culture medium, at 37°C under 5% CO_2_. Experiments were performed after the third passage after thawing. The MycoSensor qPCR Assay Kit (Agilent) was used for Mycoplasma testing, which is performed every three months as a laboratory routine.

Recombinant human IL6 was purchased from Peprotech (USA) and eluted according to the manufacturer’s instructions. HET-1A cell line was treated with different concentrations of IL6 (10, 20, and 100 ng/mL) for different periods (30 min, 24, and 48 hours). Each experiment was performed three times in triplicates.

### *In Silico* Analyses

The public data portal cBioPortal for Cancer Genomics (10, 11) was used to retrieve gene expression data from ESCC samples (n = 95). For this, the Firehose Legacy project was assessed, and Spearman correlation rho, p-values, and q-values were obtained for *IL6* mRNA levels compared to all genes from the genome. Only those correlations with q-values < 0.05 were considered statistically significant.

To evaluate whether the genes whose expression was significantly correlated with that of *IL6* were dysregulated in ESCC, previously gene expression microarray datasets (Affymetrix Human Exon 1.0 ST Array platform) generated by the group were reanalyzed (deposited in the Gene Expression Omnibus database, accession GSE75241). Differences between tumors and non-tumor surrounding tissues, as well as non-tumor surrounding tissues and healthy esophagus, were considered statistically significant when |fold-change| > 1.5 and FDR < 0.05.

The over-representation analysis was performed in the WEB-based Gene Set Analysis Toolkit (WebGestalt) using the Kyoto Encyclopedia of Genes and Genomes (KEGG) as a functional database. Only pathways with FDR < 0.05 were considered significantly enriched.

### Statistical Analyses

All statistical analyses were performed using the GraphPad Prism 5 software (GraphPad Software, USA) or R environment. When comparing two groups, unpaired t-test or Mann-Whitney were used for unpaired samples while paired t-test or Wilcoxon signed-rank tests were applied for paired samples. For determining significant differences between more than two groups, we have applied One-Way ANOVA or Kruskal Wallis test and Tukey’s post-test or Dunn’s post-test, respectively.

Receiver operating characteristic (ROC) curves were used to determine whether the proposed biomarkers were able to distinguish the sample groups.

Univariate survival analyses were carried out with the Kaplan–Meier method and Log-rank test. Gene expression cut-offs were determined according to the best performing threshold (12), being 2.53x10^-2^ *GAPDH* units for *IL6* expression and 1.24x10^-2^ *GAPDH* units for *BCL3* expression. Variables with p < 0.2 were selected for multivariate analysis. Finally, Cox regression was applied with the stepwise forward method (13). The ‘survival’ package was used.

In all cases, p values were considered statistically significant when less than 0.05.

## Results

### Characterization of the Individuals Without Esophageal Disorders and ESCC Patients Included in This Study

Among the individuals without esophageal disorders included in this study, most were female (69%), never smokers (62%), and ever drinkers (53%) (**Table 1**). This group presented a median age of 57 years (18–85), similar to what was observed in the group of ESCC patients, with a median age of 59 years (39-77). However, ESCC patients were mostly male (74%), ever smokers (98%), and ever drinkers (86%). Regarding tumors’ characteristics, most were located at the middle third of the esophagus (71%), were moderately differentiated (76%), and were mostly diagnosed in stages III and IV (71%) (**Table 1**).

### *IL6* Is Upregulated in ESCC, Produced by Tumor Cells and It Is Likely to Depict Transcriptional Programs

**Figure 1:**
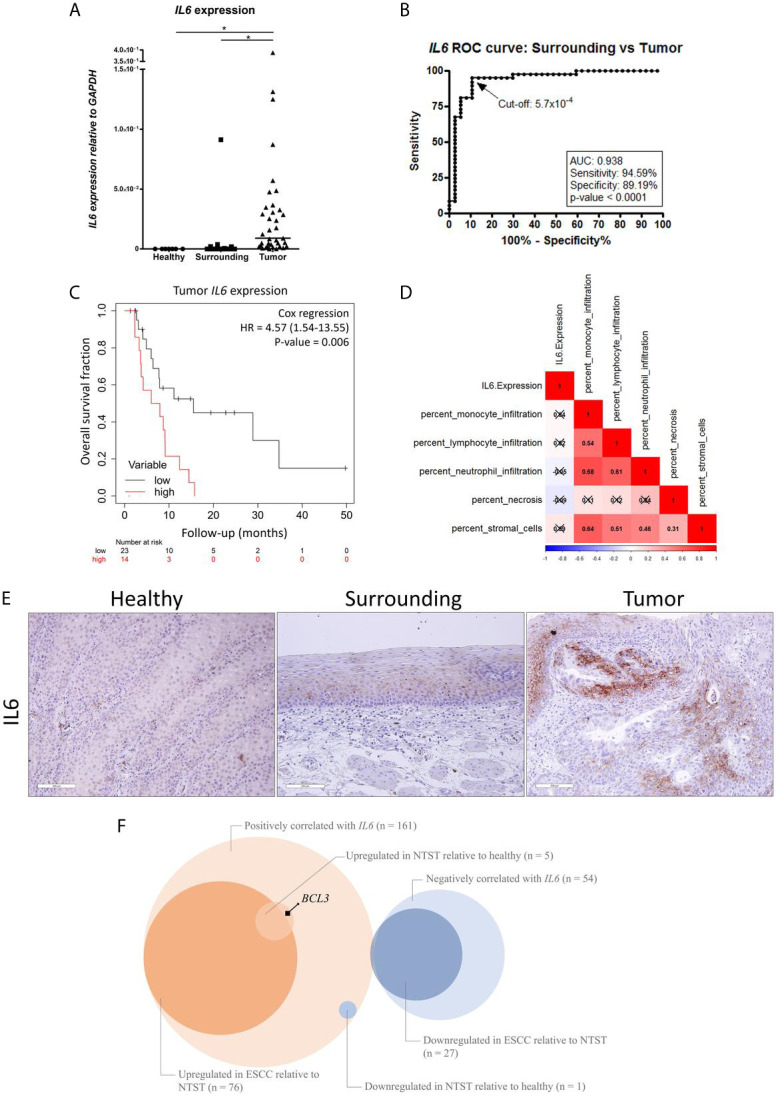
IL6 dysregulation in ESCC. **(A)** Dot-plot representing *IL6* mRNA expression normalized with *GAPDH* in esophageal mucosa from individuals without esophageal disorders (Healthy), histologically normal tumor-surrounding tissues from ESCC patients (Surrounding), and ESCC (Tumor). **(B)** Receiver Operating Characteristic (ROC) curve showing the sensitivity and specificity of *IL6* mRNA expression normalized with *GAPDH* to distinguish Surrounding and Tumor samples from ESCC patients. IL6 expression cut-off of 5.7x10-4 showed the highest accuracy (93%), sensitivity (95%) and specificity (89%) (p < 0.0001). **(C)** Kaplan-Meier curve showing the overall survival of ESCC patients according to *IL6* expression. High expression was defined as ≥ 2.53x10^-2^ *GAPDH* units, according to the best performing threshold. Tumor stage was used for adjustment. **(D)** Correlation matrix between *IL6* expression and the percentage of different cellular populations in ESCC samples from the TCGA database. Red represents positive correlations, and blue represents inverse correlations. Boxes marked with an X depict non-significant correlations (p ≥ 0.05). **(E)** Representative FFPE slides from immunohistochemistry performed with IL6 antibody in esophageal mucosa from individuals without esophageal disorders (Healthy), histologically normal tumor-surrounding tissues from ESCC patients (Surrounding), and ESCC (Tumor). Sections were counterstained with Harris’ hematoxylin. All images are shown in 20X magnification and 300 µm scale bars are shown. **(F)** Venn diagram showing the number of genes whose mRNA expression was positively or negatively correlated with *IL6* mRNA expression (q-value < 0.05) in ESCC samples from TCGA database, number of genes upregulated or downregulated in ESCC relative to non-tumor surrounding tissue (NSTS) (|fold-change| > 1.5 and FDR < 0.05) in our dataset, and number of genes upregulated or downregulated in NTST relative to healthy esophageal mucosa (|fold-change| > 1.5 and FDR < 0.05) in our dataset. *BCL3* is highlighted as a gene whose mRNA expression was positively correlated with *IL6* expression in ESCC, and that was found upregulated in NTST relative to the healthy esophagus. *p < 0.05.

We have previously shown *IL6* promoter hypomethylation in ESCC (3) and, therefore, we aimed to investigate *IL6* expression in the groups of samples included in the present work. In **Figure 1A**, we show that ESCC presents higher *IL6* mRNA levels in comparison with histologically normal esophageal samples, either from the same ESCC patients (tumor-surrounding samples, p < 0.0001), or from individuals without esophageal disorders (p < 0.0001). Furthermore, *IL6* expression was undetectable in 100% of samples from individuals without esophageal disorders, in 60% of tumor-surrounding samples from ESCC patients, but in only one (2.7%) ESCC sample. Using a Receiver Operator Characteristic (ROC) curve, we showed that *IL6* expression was able to distinguish normal-appearing surrounding tissue from tumors with an accuracy of 93%, with 95% sensitivity and 89% specificity (p < 0.0001, Cut-off = 5.7x10^-4^ *GAPDH* relative-units, **Figure 1B**). Although *IL6* mRNA levels were not associated with clinical and tumor features (**Supplementary Table 1** and **Supplementary Figure 1**), patients with high tumor *IL6* expression showed a median overall survival (OS) of 6.97 months in comparison with a median OS of 15.5 months among patients with low *IL6* expression (**Figure 1C**). Cox regression analysis further showed that high *IL6* expression is a predictor of poorer prognosis independent of tumor stage (HR = 4.57, 95% CI= 1.54-13.55, p = 0.006; **Table 2**).

**Table 2:** Overall survival analyses.

Feature	Category	Univariate Analysis			Multivariate Analysis		
		HR	95% CI	p-value	HR	95% CI	p-value
Age (years)	< 60 *vs* ≥ 60	0.91	0.42 - 1.98	0.8			
Tumor stage	III & IV *vs* I & II	2.59	0.71 - 9.44	0.1	3.51	0.90 - 13.67	0.069
Histologic grade	G3 *vs* G2	0.46	0.1 - 2.0	0.3			
*IL6* expression*	High *vs* Low	3.19	1.38 - 7.36	**0.004**	4.57	1.54 - 13.55	**0.006**
*BCL3* expression*	High *vs* Low	1.48	0.68 – 3.21	0.31			

*IL6 and BCL3 expression cut-offs were determined according to the best performing threshold, being 2.53x10^-2^ GAPDH units and 1.24 x10^-2^ GAPDH units, respectively.

HR, hazard ratio; CI, confidence interval.

Bold numbers represent p < 0.05.

Since IL6 is a cytokine that can be produced either by immune or tumor cells (14), we decided to analyze which cells were responsible for the high *IL6* expression detected in ESCC. So, we assessed the correlation between the percentage of different immune populations and *IL6* expression in ESCC using the TCGA database. This analysis revealed that there were no significant correlations between monocytes, lymphocytes, neutrophil infiltrates, or stromal cells and *IL6* expression (**Figure 1D**). Necrosis was also not correlated with *IL6* expression (**Figure 1D**). By contrast, IL6 immunohistochemistry performed in our sample set showed positive staining only in tumor cells, with some tumor areas presenting a strong cellular membrane and cytoplasmic, and intercellular staining (probably as a result of IL6 secretion) (**Figure 1E**). Complementary, we could not detect IL6 immunostaining in the esophagus from individuals without esophageal disorders, while diffuse staining was observed in tumor-surrounding esophageal mucosa (**Figure 1E**).

Next, by using TCGA database, we identified a significant correlation between the expression of *IL6* and other 215 genes in ESCC (**Supplementary Table 2**), with 161 (75%) of them presenting a positive and 54 (25%) presenting an inverse correlation (q-value < 0.05). Among the positively correlated genes, 77 (48%) were differentially expressed in ESCC when compared to the respective tumor-surrounding esophageal mucosa (|fold-change| > 1.5 and FDR < 0.05), being 76 upregulated in tumors (**Figure 1F**). When considering the 54 inversely correlated genes, 26 (48%) were downregulated in ESCC compared to the tumor-surrounding esophageal mucosa (**Figure 1F**). An over-representation analysis, including all differentially expressed genes using the KEGG database, showed that the “TNF signaling” and “*Staphylococcus aureus* infection” pathways were enriched (FDR < 0.05).

Considering the same set of 161 genes whose expression was positively correlated with *IL6* mRNA levels, six (4%) were differentially expressed in tumor-surrounding esophagus relative to esophageal mucosa from individuals without esophageal disorders (|fold-change| > 1.5 and FDR < 0.05), with only one gene found to be downregulated among the former (*ZFAND5*). In contrast, five were upregulated (*BCL3*, highlighted in the Figure, *TIMP1*, *IFITM1*, *PMP22*, and *NINJ1*) (**Figure 1F**).

**Figure 2:**
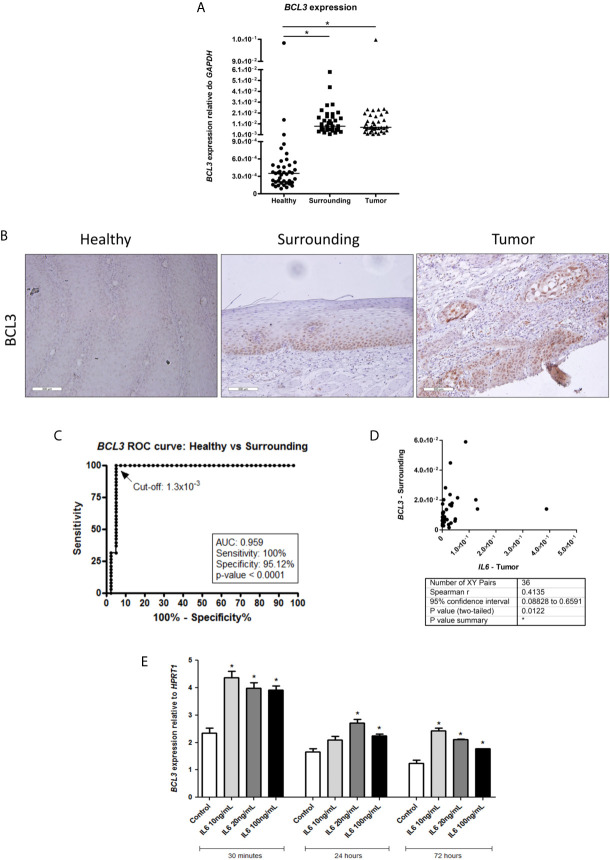
BCL3 expression in healthy esophagus and ESCC. **(A)** Dot-plot representing *BCL3* mRNA expression normalized with *GAPDH* in esophageal mucosa from individuals without esophageal disorders (Healthy), histologically normal tumor-surrounding tissues from ESCC patients (Surrounding), and ESCC (Tumor). **(B)** Representative FFPE slides from immunohistochemistry performed with BCL3 antibody in esophageal mucosa from individuals without esophageal disorders (Healthy), histologically normal tumor-surrounding tissues from ESCC patients (Surrounding), and ESCC (Tumor). Sections were counterstained with Harris’ hematoxylin. All images are shown in 20X magnification and 300 µm scale bars are shown. **(C)** Receiver Operating Characteristic (ROC) curve showing the sensitivity and specificity of *BCL3* mRNA expression normalized with *GAPDH* to distinguish Healthy from Surrounding. *BCL3* expression cut-off of 1.3x10-3 showed the highest accuracy (95.89%), sensitivity (100%) and specificity (95.12%) (p < 0.0001). **(D)** Correlation analysis between IL6 expression in tumor samples (X-axis) and *BCL3* expression in the non-tumor surrounding tissue (Y-axis) from the same patient. Each dot represents an ESCC patient. **(E)** Bar graphs showing *BCL3* mRNA expression normalized with *HPRT1* in HET-1A esophageal cells after IL6 treatment in different doses for different periods of time. *p < 0.05.

So, we decided to evaluate *BCL3* expression in our samples. **Figure 2A** shows that *BLC3* was expressed at much higher levels in tumors (~22-fold, p < 0.0001) and in normal tumor surrounding tissue (~25-fold, p < 0.0001), when compared to normal esophageal samples from patients without esophageal disorders. *BCL3* expression was not associated with etiological or clinical-pathological variables analyzed (**Supplementary Table 1** and **Supplementary Figure 1**). BCL3 protein expression was assessed by immunohistochemistry, confirming its lack of expression in esophageal epithelium from individuals without esophageal disorders. At the same time, both ESCC and tumor-surrounding mucosa showed positive nuclear staining (**Figure 2B**). We also evaluated the efficiency of *BCL3* expression to distinguish esophageal epithelium from individuals without esophageal disorders from the histologically normal mucosa from ESCC patients using a ROC curve. With a cut-off of 1.3x10^-3^, *BCL3* expression relative to *GAPDH* distinguished histologically normal samples from individuals without esophageal disorders from those with ESCC, with an accuracy of 96% (100% sensitivity and 95.12% specificity, p < 0.0001, **Figure 2C**). *BCL3* expression in the surrounding tissue was positively correlated with *IL6* expression in the tumors (Spearman r = 0.4135, p = 0.0122; **Figure 2D**).

To verify whether IL6 could modulate *BCL3* expression in the esophagus, we used a non-transformed esophageal cell line (HET-1A). We showed that this cytokine could significantly induce *BCL3* mRNA expression in all tested concentrations and time intervals, except for the treatment with 10 ng/mL for 24 hours (**Figure 2E**).

### Spatial Intratumoral Analysis for *IL6* and *BCL3* Expression

**Figure 3:**
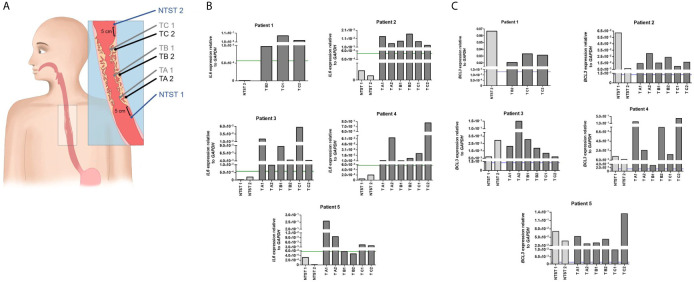
Intratumor heterogeneity of *IL6* and *BCL3* expression in ESCC. **(A)** Schematic representation of sample collection by endoscopy for the evaluation of intratumor heterogeneity. From each patient, a total of eight biopsies was collected when possible. Non-tumor surrounding tissue samples were collected 5 cm below (NTST 1) and above (NIST 2) the tumor border. ESCC samples were collected from each third of the tumor mass (lower third, TA; middle third, TB; and upper third, TC), being one superficial (1) and another profound (2), the latter collected by a biopsy-on-biopsy scheme. **(B)** Bar graphs representing *IL6* expression normalized with *GAPDH* in the different non-tumor surrounding tissue (NTST) and tumor biopsies from five patients diagnosed with ESCC (Patient 1-5). The green line represents the *IL6* expression cut-off that showed the highest accuracy to differentiate the tumor tissue from the non-tumor surrounding tissue, as shown in **Figure 1B** (5.7x10^-4^). **(C)** Bar graphs representing *BCL3* expression normalized with *GAPDH* in the different non-tumor surrounding tissue (NTST) and tumor biopsies from five patients diagnosed with ESCC (Patient 1-5). The blue line represents the *BCL3* expression cut-off that showed the highest accuracy to differentiate the non-tumor surrounding tissue from the esophageal mucosa from individuals without esophageal disorders, as shown in **Figure 2C** (1.3x10^-3^).

In order to evaluate the spatial intratumoral pattern of *IL6* and *BCL3* expression in ESCC, we used three to six tumor biopsies from different regions of the tumoral mass and two fragments of non-tumoral surrounding tissue, from five patients with ESCC (**Figure 3A**). Corroborating the results presented in **Figure 1A**, *IL6* overexpression was observed in all tumoral fragments of all patients, compared to their respective non-tumoral surrounding tissues (**Figure 3B**). Only in two tumor fragments from Patient 5, *IL6* expression did not surpass the previously established cut-off for differentiating tumor and non-tumor surrounding tissue (using the ROC curve cut-off = 5.7x10^-4^ *GAPDH* relative-units) (**Figure 3B**). Spatial intratumoral heterogeneity was observed for *IL6* expression within the tumor mass in four out of five patients. In addition, using the previous cut-off of 1.3x10^-3^ *GAPDH* relative-units defined to distinguish morphologically normal tissues from healthy individuals and ESCC patients, *BCL3* expression exceeded this cut-off in every analyzed fragment of tumoral and non-tumoral tissues (**Figure 3C**).

## Discussion

IL6 is a pleiotropic cytokine upregulated in different tumor types, and it can depict several transcriptional programs. Here we propose IL6 produced by ESCC tumors cells might induce the expression of several genes and downregulate a number of others in a juxtacrine fashion, but also induce the expression of genes involved in key cancer-associated pathways in the non-tumor surrounding tissue. One of these targets might be *BCL3*, a recognized oncogene for hematological malignancies, whose alterations have been associated with tumor progression and poor prognosis (15, 16). More recently, the role of *BCL3* in solid tumors started to be elucidated, and its overexpression, observed in breast and nasopharyngeal tumors, seems to be induced by NF-κB activation and EBV latent genes, respectively (17–19). Here, we show for the first time *BCL3* upregulation in esophageal squamous cell carcinoma and its high potential as a diagnostic biomarker, since it precedes the first histopathological alterations.

A key aspect of esophageal carcinogenesis is the development of synchronic tumors that has been attributed to field cancerization. This concept was first proposed by Slaughter and colleagues (20) to explain a similar phenomenon observed in oral carcinogenesis and assumes that the entire organ, in this case, the entire esophagus could present patches of premalignant cells that, although may not show any morphological alteration, already carry molecular alterations that predispose to neoplastic transformation. In this context, *BCL3* overexpression in the normal-appearing tumor-surrounding tissue observed in this study could represent one of these predisposing molecular alterations. It has been proposed before by us and other authors that epigenetic alterations, specifically alterations of DNA methylation, could be the first disturbance to occur during the formation of the cancerization field in the esophagus (3, 4, 21). Recently, we have shown the great potential of *TFF1* expression downregulation, one of the genes epigenetically deregulated, as an early ESCC detection biomarker, since it takes place in the tumor-surrounding tissue from ESCC patients, preceding the first morphological and genetic alterations (22).

Along with these findings, we demonstrated that *BCL3* expression is able to distinguish healthy esophagus from non-tumor tissue adjacent to ESCC with high accuracy, sensitivity, and specificity and could help to identify the patches of premalignant esophageal cells. Moreover, using the proposed cut-off for mRNA analysis, *BCL3* overexpression was consistent in non-tumor adjacent tissues and tumors in the spatial intratumoral analysis, showing its robust potential as a biomarker of diagnosis. This could be particularly useful in patients with head and neck cancer since these individuals show a relatively high incidence of second primary tumors in the esophagus, impacting their overall survival (23). Another important point to discuss is the currently available methods for ESCC early diagnosis. Although the impact of downstaging through screening on patient mortality is clear, there is no globally accepted method. Endoscopy following lugol staining (chromoendoscopy) is one of the most commonly used approaches and shows high sensitivity, but low specificity (24). Therefore, molecular alterations that are detectable before morphological changes and are maintained during tumor development (independently of tumor stage) can be useful in helping to identify those individuals at risk, potentially increasing specificity of the screening methods.

In the present study, we also observed a positive correlation between *IL6* expression in tumors and *BCL3* expression in tumor-adjacent samples of ESCC patients, suggesting a possible dysregulation of this axis in the early stages of esophageal carcinogenesis. It has been shown before that IL6 is capable of inducing *BCL3* transcription in multiple myeloma cells *via* STAT3 binding to an enhancer in the *BCL3* gene body (24). Since *IL6* overexpression is only observed in ESCC samples, while *BCL3* higher mRNA levels are detected both in tumor-surrounding mucosa and esophageal tumors, paracrine IL6 signaling could be responsible for *BCL3* induction in tumor-adjacent tissue. In agreement with this hypothesis, we have shown that this cytokine can induce *BCL3* expression in immortalized esophageal cells. However, this inflammatory pathway may not be the only mechanism involved in *BCL3* regulation in ESCC tissues, and further studies should be performed to confirm this hypothesis.

BCL3 has been shown to contribute to carcinogenesis through different mechanisms and mediators. Although most studies have shown that BCL3 promotes cell survival and proliferation of different cell types for its role as a key regulator of NF-κB signaling (25, 26), recent works have shown that BCL3 contribution to cancer goes beyond this role in these two cancer hallmarks. So, in mouse embryonic stem cell (mESCs), BCL3 seems to regulate proliferation and pluripotency by influencing Nanog transcription (27). A similar effect has been observed in colorectal cancer cells, in which BCL3 acts as a co-activator of β-catenin/TCF-mediated transcriptional activity and induces the expression of stemness markers such as *LGR5* (Leucine-Rich Repeat Containing G Protein-Coupled Receptor 5) and *ASCL2* (Achaete-Scute Family BHLH Transcription Factor 2) (28). Other BCL3 transcriptional targets include *PD-L1* (Programmed Cell Death 1 Ligand 1) and *CAII* (Carbonic Anhydrase II) that mediate cell proliferation and resistance to alkylating agents in ovarian cancer and gliomas, respectively (29, 30). Therefore, *BCL3* overexpression before esophageal transformation may contribute not only to apoptosis inhibition and induced proliferation capacity, but also to the acquisition of stemness phenotypes. The maintenance of its high expression levels in tumors should also be further investigated in terms of immune escape and resistance to therapy.

Our study showed a consistent *IL6* overexpression in all ESCC patients, and the ability to distinguish the surrounding mucosa from tumoral tissue with high sensitivity and specificity. *IL6* upregulation in esophageal cancer has been shown before by other authors, and it has been proposed that serum levels of this cytokine could be used as a diagnostic biomarker in ESCC, with higher efficiency than classic tumor markers (carcinoembryonic and squamous cell cancer antigens), and could also predict overall and disease-free survival (31–33). Our data corroborated the association of high *IL6* expression with a poor prognosis in ESCC. Also, it has been proposed that IL6 could be a therapeutic target in ESCC. Currently, two drugs targeting IL6 signaling are clinically registered, siltuximab, an anti-IL6 mAb, and tocilizumab, an anti-IL6R mAb. Although the FDA approved the treatment of patients with multicentric Castleman disease with siltuximab (34), the studies in cancer patients are still scarce.

Early diagnosis of esophageal squamous cell carcinoma has not improved in recent years, indicating not only a lack of knowledge on the mechanisms of early alterations and development of ESCC, but also the difficulty in identifying and validating robust biomarkers of early tumor detection. According to data from us and other authors (3, 4, 35–37), inflammation seems to be an essential hallmark of ESCC and may provide essential biomarkers to anticipate diagnosis, predict prognosis, and may represent new therapeutic targets.

In conclusion, our study shows the consistent *IL6* overexpression in ESCC cells, suggesting its potential use as a therapeutic target for ESCC, and the paracrine induction of the expression of the antiapoptotic gene *BCL3*, revealing alterations that may contribute to ESCC development. Furthermore, our study highlights a set of data to support the use of *BCL3* mRNA expression as a biomarker of ESCC detection, suggesting further studies should be performed to corroborate these findings in high-risk groups for ESCC development, as head and neck cancer patients.

## Data Availability Statement

The datasets presented in this study can be found in online repositories. The names of the repository/repositories and accession number(s) can be found below: https://www.ncbi.nlm.nih.gov/geo/, GSE75241 https://www.cbioportal.org/study/summary?id=esca_tcga, Esophageal Carcinoma (TCGA, Firehose Legacy).

## Ethics Statement

The studies involving human participants were reviewed and approved by Comissão Nacional de Ética em Pesquisa - CAAE 0086.0.007.000-11 Comitê de Ética em Pesquisa do Instituto Nacional de Câncer - CEP n° 116/11. The patients/participants provided their written informed consent to participate in this study.

## Author Contributions

Conceptualization, ZH, HH-V, LR, and SS-L. Methodology, IG, SG, HH-V, and SS-L. Validation, IG, PN-N, and RV. Formal analysis, IG, DC, PN-N, RV, and SS-L. Investigation, IG, DC, MF, SG, and RV. Resources, ZH, LP, and SS-L. Data curation, IG, DC, and SS-L. Writing—original draft preparation, IG, DC, and SS-L. Writing—review and editing, IG, DC, PN-N, RV, MF, SG, HH-V, ZH, LR, and SS-L. Visualization, IG, DC, RV, HH-V, ZH, LR, and SS-L. Supervision, LR and SS-L. Project administration, LR and SS-L. Funding acquisition, LR, ZH, and SS-L. All authors contributed to the article and approved the submitted version.

## Funding

This research was funded by the Fundação de Amparo à Pesquisa do Estado do Rio de Janeiro (FAPERJ) grant E-26/010.001856/2015 and the Conselho Nacional de Desenvolvimento Científico e Tecnológico (CNPq) grant 407992/2016-2.

## Author Disclaimer

Where authors are identified as personnel of the International Agency for Research on Cancer/World Health Organization, the authors alone are responsible for the views expressed in this article and they do not necessarily represent the decisions, policy or views of the International Agency for Research on Cancer/World Health Organization.

## Conflict of Interest

The authors declare that the research was conducted in the absence of any commercial or financial relationships that could be construed as a potential conflict of interest.

## Publisher’s Note

All claims expressed in this article are solely those of the authors and do not necessarily represent those of their affiliated organizations, or those of the publisher, the editors and the reviewers. Any product that may be evaluated in this article, or claim that may be made by its manufacturer, is not guaranteed or endorsed by the publisher.

## Supplementary Material

Supplementary Figure 1 *IL6* and *BCL3* expression association with tumor staging and impact on overall survival. Dot plots representing *IL6* and *BCL3* expression relative to *GAPDH* according to T **(A)**, N **(B)** and M **(C)** from TNM staging system.
