# Supplementary figures and images for: IL6 and BCL3 Expression Are Potential Biomarkers in Esophageal Squamous Cell Carcinoma
Source: Front Oncol. 2021 Aug 4;11:722417. doi: 10.3389/fonc.2021.722417 (PMC8371528; doi:10.3389/fonc.2021.722417)

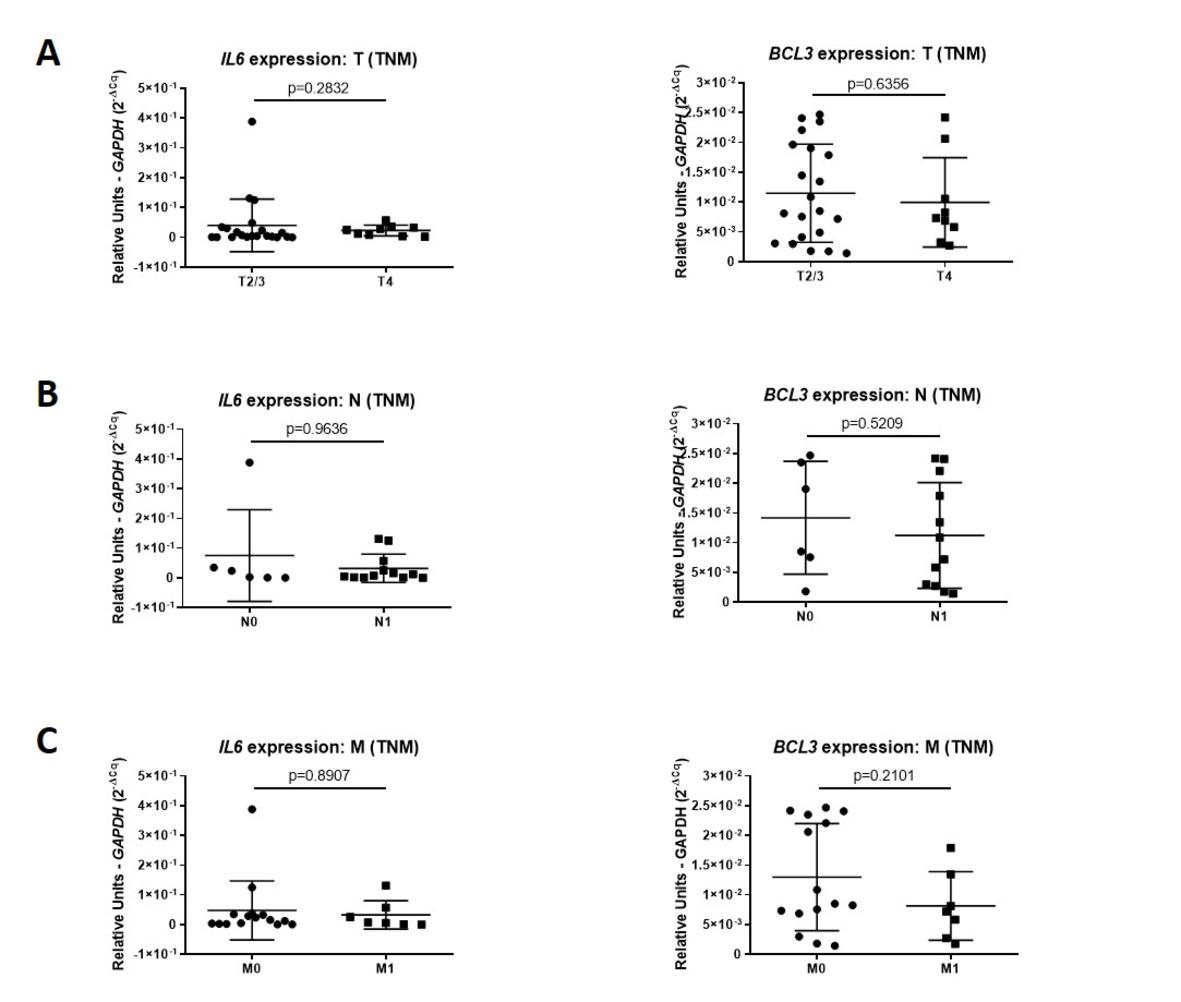

Supplement: Supplementary Figure 1 — IL6 and BCL3 expression association with tumor staging and impact on overall survival. Dot plots representing IL6 and BCL3 expression relative to GAPDH according to T (A), N (B) and M (C) from TNM staging system. [file Image_1.jpeg]
